# Accuracy of Treatment Recommendations by Pragmatic Evidence Search and Artificial Intelligence: An Exploratory Study

**DOI:** 10.3390/diagnostics14050527

**Published:** 2024-03-01

**Authors:** Zunaira Baig, Daniel Lawrence, Mahen Ganhewa, Nicola Cirillo

**Affiliations:** 1 Melbourne Dental School, The University of Melbourne, 720 Swanston Street, Carlton, VIC 3053, Australia; 2 CoTreat Pty Ltd., Melbourne, VIC 3000, Australiamax.ganhewa@cotreat.com.au (M.G.); 3 School of Dentistry, University of Jordan, Amman 11733, Jordan

**Keywords:** evidence-based guidelines, treatment recommendations, Artificial Intelligence, ChatGPT, Bard

## Abstract

There is extensive literature emerging in the field of dentistry with the aim to optimize clinical practice. Evidence-based guidelines (EBGs) are designed to collate diagnostic criteria and clinical treatment for a range of conditions based on high-quality evidence. Recently, advancements in Artificial Intelligence (AI) have instigated further queries into its applicability and integration into dentistry. Hence, the aim of this study was to develop a model that can be used to assess the accuracy of treatment recommendations for dental conditions generated by individual clinicians and the outcomes of AI outputs. For this pilot study, a Delphi panel of six experts led by CoTreat AI provided the definition and developed evidence-based recommendations for subgingival and supragingival calculus. For the rapid review—a pragmatic approach that aims to rapidly assess the evidence base using a systematic methodology—the Ovid Medline database was searched for subgingival and supragingival calculus. Studies were selected and reported based on the Preferred Reporting Items for Systematic Reviews and Meta-analyses (PRISMA), and this study complied with the minimum requirements for completing a restricted systematic review. Treatment recommendations were also searched for these same conditions in ChatGPT (version 3.5 and 4) and Bard (now Gemini). Adherence to the recommendations of the standard was assessed using qualitative content analysis and agreement scores for interrater reliability. Treatment recommendations by AI programs generally aligned with the current literature, with an agreement of up to 75%, although data sources were not provided by these tools, except for Bard. The clinician’s rapid review results suggested several procedures that may increase the likelihood of overtreatment, as did GPT4. In terms of overall accuracy, GPT4 outperformed all other tools, including rapid review (Cohen’s kappa 0.42 vs. 0.28). In summary, this study provides preliminary observations for the suitability of different evidence-generating methods to inform clinical dental practice.

## 1. Introduction

A key pillar of dental practice is the provision of quality care to improve a patient’s oral health. However, in order to do so, healthcare must be underpinned by evidence. Evidence-based guidelines (EBGs) ensure that clinical decisions and treatments are reflective of the current literature [1]. Yet, there is limited availability of high-quality EBGs in the field of dentistry [1,2]. Clinical practice is significantly based on expert opinion, whereas EBGs allow empirical evidence to be primarily implemented [2]. Moreover, standardized diagnoses for dental conditions are imperative. Many dental diseases have several diagnostic criteria existing in the literature; not only does this introduce variation but it also hinders the diagnostic workflow. Optimal patient management requires EBGs, which include the most substantiated diagnostic criteria with evidence-based treatment options. Conversely, certain conditions have limited literature on their diagnostic criteria, such as secondary caries compared to primary free smooth surface caries [3]. There is also a demand to research diagnostic tools that may be implemented in teledentistry, instead of solely relying on clinical oral examinations [4]. In recent times, coronavirus disease 2019 (COVID-19) has particularly highlighted such a need. Thus, clinical EBGs that allow photographic and radiographic diagnoses of dental conditions, with corresponding treatment recommendations, must be created. This will improve teledentistry outcomes by providing a framework for treatment planning. Furthermore, the emergence of generative Artificial Intelligence (AI) programs has instigated interest in its application in dentistry. Large language models (LLMs), such as ChatGPT, from OpenAI, and Bard, from Google AI, have gained significant popularity [5]. The potential of natural language processing AI for clinical decision making must be explored [6]. The concept of AI has been introduced in all disciplines of dentistry, ranging from endodontics, with AI detection of periapical lesions, root canal fractures, and morphology, to prosthodontics, with restorative designing through CAD/CAM systems [7]. In general practice, deep learning can automate processes, such as detecting the number of teeth on radiographs and facilitating radiographic diagnoses of carious lesions and periodontal bone loss [7,8]. Surveys also suggest that most patients have positive attitudes towards the introduction of AI in their oral health experience [9]. With the increased burden on public and private dental systems, EBGs can streamline the provision of oral healthcare and accelerate advancement in remote and AI-augmented recommendations [10]. 

The aim of this pilot study was to develop a protocol to reliably create EBGs for dental conditions, which may be implemented by dental practitioners in the clinical setting, teledentistry, and used as criterion guidelines for research purposes. The current literature includes significant evidence to support and facilitate treatment guidelines for supragingival and subgingival calculus, and the preliminary results show that human findings are comparable to outcomes suggested by AI.

## 2. Materials and Methods

### 2.1. Study Workflow and Evidence Generating Tools

Dental calculus was selected as a test item as it can be observed through intraoral photographs or radiographs (periapical radiograph (PA), bitewing (BW) radiograph, or orthopantomagram (OPG)). A rapid systematic review was carried out on Ovid MEDLINE using a predefined search strategy. A search of Artificial Intelligence (AI) chatbots, including ChatGPT3.5 and GPT4 by OpenAI (San Francisco, CA, USA) and Bard (currently rebranded as Gemini) by Google AI (Mountain View, CA, USA), with specific inputs regarding treatment recommendations, was also undertaken. The reference standard was generated by CoTreatAI (Melbourne, VIC, Australia) using a Delphi approach. Treatment recommendations were then compared using qualitative and quantitative content analysis tools.

### 2.2. Delphi Group and Guideline Development

The Delphi group was made up of 6 panelists: 5 dentists who were tasked with defining calculus and developing evidence-based treatment recommendations and 1 member with experience in clinical governance and leadership in healthcare (D.L.). 

Panelists for the Delphi group were carefully chosen to ensure a diverse selection of dentists (two females and three males) with a breadth of experience in both private practice and academia, averaging 15 years. To mitigate dominance bias during face-to-face discussions, a non-dentist with expertise in management and leadership was appointed as the moderator. In the Delphi process’s initial round, treatment proposals were independently and anonymously drafted to minimize groupthink and conformity biases. These initial recommendations were subsequently deliberated in a meeting, culminating in a consensus. For the second round, each panelist was charged with the task of identifying common themes within the treatment guidelines. A consensus on the final list of themes was achieved during a face-to-face meeting. On the rare occasions where disagreement arose, issues were resolved through anonymous majority voting on proposals put forth by the moderator, which were based on the panelists’ suggestions.

Based on a systematic search (Supplementary Table S1 and Figure S1) and discussion, the following definitions were used to inform the search for treatment recommendations:

1. *Supragingival calculus (tartar)*, defined as a mineralized byproduct of bacterial plaque deposits that form on the surfaces of teeth above the gingival margin and can be whitish to yellowish in color.
2. *Subgingival calculus*, defined as radiopaque irregularity at cementoenamel junction or extending beyond or superimposed over the root surface contour on the PA or BW radiograph.

As per group consensus among the Delphi group, definitions were developed such that these conditions can be identified through the use of imaging. Thus, they are suitable for teledentistry and/or AI processing. 

Regarding the development of treatment guidelines, the Delphi group focused on high-impact clinical observations and recommendations that were deemed to be evidence-based and a necessity to ensure patient harm prevention.

### 2.3. Gathering Treatment Recommendations through a Rapid Systematic Review

#### 2.3.1. Search Strategy

One investigator (Z.B.) used a pragmatic review approach—the rapid review—as this methodology adapts the conventional systematic review process to take into consideration the limited time and/or resources available. Hence, as a rapid review is typically used when there are time constraints, this approach more realistically replicates a clinician’s search for evidence in a practice setting. A search of the Ovid MEDLINE database for the following categories with specific search strings for each category and their respective treatment was conducted (Table 1). The Preferred Reporting Items for Systematic Review and Meta-Analyses (PRISMA) protocols were used, and this study complied with the minimum requirements for completing a “restricted systematic review” [11]. 

#### 2.3.2. Eligibility Criteria

Studies were eligible if they met the following inclusion criteria: (a) the specified diagnostic criteria were tested using either photographic or radiographic means or (b) tested treatment options based on the same diagnostic classification. For each category, articles with the highest level of evidence were collated. 

Non-English articles, reviews, books, and letters were excluded. Animal studies were also excluded. There was no publication year restriction. 

#### 2.3.3. Data Collection and Quality Assessment

The selection process was conducted by one reviewer. In the initial phase, title screening was conducted based on the inclusion and exclusion criteria. In the second phase, titles and abstracts were screened. In the final phase, full-text article readings were conducted and selected based on the inclusion criteria. Duplicate articles were removed. Articles were only selected if they utilized the specified diagnostic classification or had corresponding treatment outcomes. For each category, from the pool of eligible articles, the article(s) with the highest grade of evidence were included for the formation of the EBGs. 

### 2.4. Treatment Recommendations Provided by Artificial Intelligence

Regarding AI, specific search inputs were placed in a new chat for each condition in ChatGPT and Bard (Table 2). The AI model was not prompted further, and the recommendations provided were summarized. Supragingival and subgingival calculus were strictly defined to ensure only observations through either photographs or radiographs were included.

### 2.5. Qualitative and Quantitative Content Analysis

The comparative analysis involved three main steps: identifying criteria for comparison, categorizing responses from each source regarding these criteria (qualitative content analysis, QCA), and calculating the rate of agreement (quantitative analysis). Criteria were derived from the provided text and encompassed practices, such as professional mechanical plaque removal, tool usage, and adjunctive treatments. Each source was reviewed to determine whether it agreed with (displayed as green), disagreed with (red), did not mention the criteria (yellow), and had partial or conditional agreement/disagreement (orange). The agreement rate for each criterion was calculated using Cohen’s k score (when assessing the agreement between two tools) and Fleiss’ kappa (for assessing the reliability between multiple tools). Note that when calculating kappa scores, partial/conditional agreement was coded as agreement to overcome statistical errors.

## 3. Results

### 3.1. Development of Evidence-Based Guidelines

**Figure 1:**
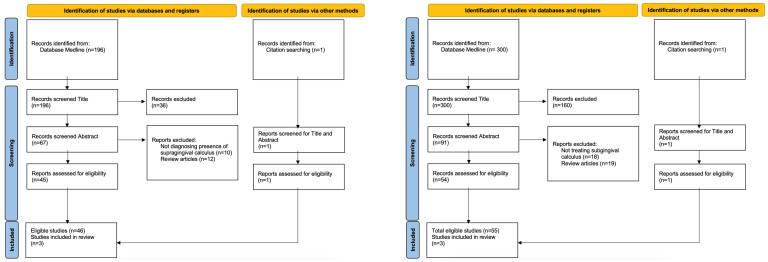
Flowchart of the study selection process for rapid review included searches of databases, registers, and other sources for supragingival (**left**) and subgingival (**right**) calculus, according to the Preferred Reporting Items for Systematic Reviews and Meta-Analyses (PRISMA) guidelines.

The Delphi group panelists undertook an independent review of systematic reviews and professional guidelines, and seven themes as a framework for evaluation were proposed following open discussion (see Section 3.4). For the rapid review by the individual clinician, treatment guidelines were scoped for supragingival and subgingival calculus from Ovid Medline. As of 6 June 2023, our search strategy yielded a total of 498 hits (Figure 1). For supragingival calculus, three treatment studies were included from a total of 197 articles. This includes one clinical practice guideline and two randomized controlled trials (RCTs) (Supplementary Table S2) [12,13,14]. 

For subgingival calculus, three treatment studies were included from a total of 301 articles. This includes one clinical practice guideline and two systematic reviews [12,15,16]. Two studies found that adjuncts, including antimicrobial therapy and photodynamic therapy, did not facilitate subgingival calculus removal (Supplementary Table S3) [12,16].

### 3.2. Overview of Treatment Recommendations for Supragingival Calculus

All tools acknowledged the importance of core elements, such as routine professional mechanical removal and oral hygiene. However, the procedure was referred to as scaling and root planning by GPT3.5 and Bard, whereas the rapid review correctly identified this item as professional mechanical plaque removal (PMPR) for the removal of supragingival calculus. The use of hand scalers, curettes (like the universal curettes 4L–4R), and ultrasonic scalers for removing calculus was also recommended, with few exceptions (Appendix A). Each approach emphasized the need to educate patients on proper oral hygiene practices to prevent the recurrence of plaque and calculus.

Discrepancies were seen for the additional themes, particularly for adjunctive methods. For example, the rapid review recommended the use of Er:YAG lasers to reduce plaque levels, whereas Delphi advised against replacing conventional PMPR with adjunctive methods, like lasers or photodynamic therapy. GPT4 and GPT3.5 did not mention lasers but suggested adjunctive treatments, like fluoride treatments and antimicrobial mouth rinses, but none of these have a clear evidence base.

Risk factor assessment and management was explicitly mentioned in the rapid review and by GPT4, only briefly touched on by GPT3.5, and not mentioned by Bard. This indicates that recommendations on wider management practices may need to be specifically prompted for AI. 

### 3.3. Overview of Treatment Recommendations for Subgingival Calculus

All approaches recommend subgingival periodontal instrumentation as a key treatment for periodontitis in the form of scaling and root planning (SRP), which is universally recognized as the primary or gold standard treatment for removing subgingival calculus.

There was overall consensus that SRP can be performed with hand instruments, such as Gracey curettes, as well as ultrasonic scalers, but no specific recommendations were offered. Also, Bard did not provide information on instrumentation. Oral hygiene instruction was emphasized across most sources as an integral part of periodontal therapy. There was potential disagreement regarding adjunctive treatments, although these were classified as orange given that locally administered antiseptics and antimicrobials may be considered in specific cases. The Delphi group was cautious about adjunctive therapies, recommending against the use of lasers, photodynamic therapy, and several pharmacological agents as adjuncts to subgingival instrumentation.

### 3.4. Qualitative Content Analysis

**Figure 2:**
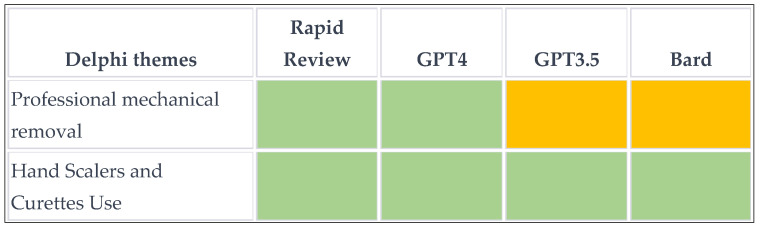

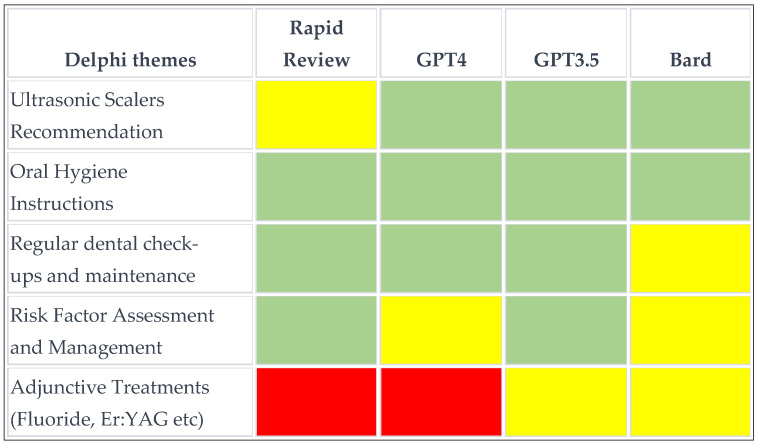
Heatmap of the agreement on 7 themes by the different tools on the treatment of supragingival calculus. *Green*, agreement; *red*, disagreement; *yellow*, not reported, *orange*, conditional agreement.

**Figure 3:**
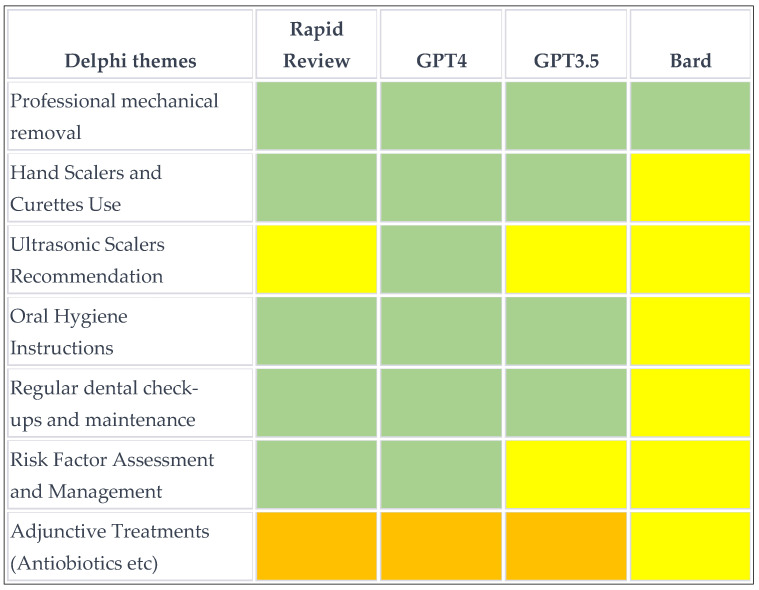
Heatmap of the agreement on 7 themes by the different tools on the treatment of subgingival calculus. *Green*, agreement; *red*, disagreement; *yellow*, not reported; *orange*, conditional agreement.

The Delphi group individuated four core themes (mechanical removal, use of hand scalers and curettes, recommendation of ultrasonic scalers, and oral hygiene instruction) and three additional themes (regular dental check-ups and maintenance, risk factor assessment and management, and adjunctive treatments, such as fluoride, mouth rinses, antibiotics). The heatmap in Figure 2 and Figure 3 show a substantial agreement for the four core themes, whereas the additional items were inconsistently reported. It is worth noting that there was a conflict in the recommendation of Er:YAG lasers as adjuncts between the Delphi and the solo rapid review. None of the AI could provide source information, although Bard made references to the published literature.

### 3.5. Quantitative Content Analysis 

Agreement for key procedures across all tools was remarkable (75% for supragingival calculus and 69% for subgingival calculus), whereas this dropped to 41.67% for additional procedures. Overall, the agreement rate was 60.7% when applying strict criteria for agreement (i.e., conditional agreement considered as non-agreement). However, it must be noted that a considerable part of the disagreement was related to missing rather than incorrect output. When comparing the treatment-generating tools individually against the reference standard, GPT4 outperformed solo rapid review (agreement with Delphi of 85.7% vs. 78.6%, Cohen’s kappa 0.416 vs. 0.276, considering partial/conditional agreement as “agreement”), followed by GPT3.5 and Bard. The Fleiss’ kappa score for all comparators was 0.118, indicating that the agreement among these evidence-generating tools is overall only slightly beyond what would be expected by chance. This increased to 0.234 when Bard was excluded. The internal agreement among “Rapid Review”, “GPT4”, and “GPT3.5” was fair (Fleiss’ kappa = 0.317). This suggests considerable heterogeneity in the output of individual tools.

## 4. Discussion

The present study examined the accuracy of EBGs developed via rapid systematic review or through generative AI models. The results suggest that generative AI may be a valid alternative for treatment recommendation in dental practice settings. 

The development of standardized diagnostic criteria associated with evidence-based treatment options is integral to the practice of dentistry. The creation of these EBGs is based on cited recommendations, which empower clinicians to make informed decisions for their patients. The application of these EBGs in teledentistry was prioritized. Treatment recommendations suggested by AI generally aligned with the literature. Bard cited systematic reviews and studies for supragingival calculus [17]. In contrast, ChatGPT did not cite the literature [18]. In this study, AI was not prompted further to provide evidence to ensure standardized methodology. Additional engagement with AI may be considered for future studies.

Supragingival calculus is defined as calcified deposits that can be visually detected [12]. Management includes removal using instrumentation, risk factor analysis, and oral hygiene instruction [12,13]. An existing clinical practice guideline, established by the European Federation of Periodontology (EFP), was used. This article assessed the quality of evidence and provided graded recommendations and strength of consensus. These are based on systematic reviews, the highest level of evidence, and RCTs. AI produced similar treatment options, emphasizing the use of scalers to remove supragingival calculus [17,18]. Bard cited the EFP guidelines as the basis of their recommendations, denoting a commonality between the rapid review and AI [14]. However, variation existed in their recommendations for adjunctive treatment and risk factor management compared to the rapid review [17,18]. 

Subgingival calculus exists below the marginal gingiva and may require radiographic detection. The EFP clinical practice guidelines and systematic reviews were used to determine recommendations. This includes mechanical removal with instrumentation [12,15]. It was noted that this debridement may be associated with root surface cementum removal [12]. There was no evidence to suggest the use of adjuncts, including antimicrobials and photodynamic therapy [12,16]. Systematic reviews are deemed to be of the highest level of evidence. However, it must be considered that both rapid reviews included a limited number of articles, which reduces the sample size from which conclusions can be drawn [15,16]. AI had recommendations in agreement with the rapid review in terms of treatment and risk factor management. However, the rapid review and Bard advised against the use of adjuncts, whereas GPT4 and GPT3.5 advocated for its implementation [17,18]. Overall, the Delphi group followed the EFP guidelines while integrating them with other evidence with the primary aim of preventing unnecessary treatments and patient harm. This approach may have highlighted the observed tendency of other evidence-generating tools to include treatments that are not supported by a strong evidence base. In this regard, it must be noted that the level of heterogeneity in the results returned by different AI tools was substantial. This level of heterogeneity signifies that there may be underlying differences in how each tool interprets or analyses the data, which could be due to distinct algorithms, decision thresholds, or conceptual frameworks employed by each tool. The presence of heterogeneity is significant because it suggests that these tools are not interchangeable and may offer diverse perspectives or conclusions.

Artificial Intelligence is being experimented primarily for diagnostic purposes. A systematic review has shown that photographic examination and subsequent image analysis provide comparable accuracy to visual inspection for the diagnosis of common dental conditions [19], including dental calculus [20]. There are, however, major limitations, both technical and medico-legal, to the use of AI in the diagnostic process [21]. Conversely, treatment guidelines have been comparatively less explored. Although diagnostic observations can facilitate clinical advancements [22] and decision making under uncertainty [23], treatment recommendations must be underpinned by published evidence. However, the evidence that informs best practice is not always readily available due to barriers to open access [24] and may not be accessible to clinicians. We reasoned that with ever-increasing treatment recommendations, dentists can use AI to generate treatment plans. This is particularly relevant to solo clinicians, as we have shown that they are more likely to change their minds on treatment plans when confronted with the results of collective intelligence [25].

However, a major limitation of accessing EBGs in the traditional way is the difficulty in application to a practitioner’s daily workflows. It is impractical for practitioners to cross-check every treatment plan against relevant EBGs in a busy clinical practice. In future, AI-powered platforms could connect dentist-made treatment plans to EBGs automatically to help fill this void. Although this was a pilot study aimed at developing a suitable protocol, rather than providing accurate comparisons, the results show that AI-based tools have remarkable potential for routine clinical use. 

Although this study was exploratory and aimed to establish a procedure for comparing the accuracy of treatment guidelines between human and machine outputs, it is important to recognize certain methodological limitations. The small sample size of themes could impact the reliability of Cohen’s kappa and Fleiss’ kappa values. Additionally, this study’s internal and external validity may also have limitations. The Delphi group was built following criteria previously outlined by our group [25]. While the size of this Delphi group may be regarded as small, published studies in health applications have used panel sizes from as low as four individuals, bearing in mind that size “should be governed by the purpose of the investigation” [26]. Also, collective intelligence benefits from anonymous decision making and, accordingly, treatment recommendations, were drafted independently, and only the consensus treatment was obtained via open discussion. This discussion was facilitated by a non-dentist to mitigate dominance bias. Nevertheless, the Delphi process is prone to bias, which may have limited the internal validity of the results. 

The development of these EBGs was not without limitations in terms of external validity. Chiefly, the rapid review was undertaken by one reviewer only. Although there was no publication date restriction, only English articles were included, which may introduce publication bias. The studies included had higher heterogeneity with variation in methodology and inclusion criteria, creating difficulty in making study comparisons. Furthermore, not all studies included a long-term follow-up on their proposed clinical interventions. This reduces the clinical applicability of treatment options. The inclusion of cross-sectional studies and case reports may have also reduced the strength of evidence. However, their inclusion was necessary to reduce publication bias and, in such cases, no other eligible studies were found in the literature. This highlights the need for the development of robust RCTs with long-term monitoring. Overall, there is a need for larger studies with detailed methodology and standardized protocols to better support treatment recommendations.

## 5. Conclusions

There have been significant advances in the field of dentistry, with the emerging literature aiming to optimize clinical practice. In this pilot study, EBGs were designed for dental practitioners that include diagnostic criteria and corresponding treatment options for subgingival and supragingival calculus. The results show that treatment recommendations by AI programs, including ChatGPT and Bard, generally aligned with the literature, particularly with the results of the clinician’s rapid review. AI has the potential to increase clinician confidence, improve efficiency, and optimize treatment planning; hence, this field of application should be explored thoroughly. Further research is required on its applicability and integration into dentistry. 

## Figures and Tables

**Table 1:** Search strategy for the rapid systematic review.

Dental Condition	Search Strategies
Supragingival calculus	(“supragingival calculus” or (“supragingival” and “calculus”)) and (“treat*” or “manag*” or “periodontitis treatment” or “remov*” or “reduc*”)
Subgingival calculus	(“subgingival calculus” or (“subgingival” and “calculus”)) and (“treat*” or “manag*” or “periodontitis treatment” or “remov*” or “reduc*”)

**Table 2:** Artificial Intelligence (AI) search strategies in ChatGTP and Bard.

Dental Condition	AI Search Strategy
Supragingival calculus	For dentists, provide evidence-based guidance for the treatment of supragingival calculus. The presence of supragingival calculus was observed through photographs.
Subgingival calculus	For dentists, provide evidence-based guidance for the treatment of subgingival calculus (presence of radiopaque irregularity at the cementoenamel junction or extending beyond or superimposed over the root surface contour on periapical or bitewing radiograph).

## Data Availability

Data are available upon request from the corresponding author.

## Supplementary Materials

The following supporting information can be downloaded at https://www.mdpi.com/article/10.3390/diagnostics14050527/s1, Table S1: Search Strategy from Ovid Medline. Table S2: Supragingival Calculus. Table S3: Subgingival calculus. Figure S1: PRISMA 2020 for systematic reviews, which included searches of databases, registers and other sources for the diagnosis of supragingival calculus and subgingival calculus.

## Appendix A

**Table A1:** Tabulated outputs for the treatment of supragingival calculus according to five tools, namely, Delphi group, rapid review, ChatGTP4, ChatGTP3.5, and Bard.

Delphi group (CoTreat AI)	Professional mechanical plaque removal (PMPR): removal of the supragingival dental biofilm and calcified deposits (calculus). Procedures: Professional mechanical plaque removal (PMPR). Delphi suggests performing routine PMPR, either as a part of a supportive periodontal care program [9] or as a routine treatment for the removal of supragingival calculus. There is insufficient evidence to recommend adjunct treatments; EFP suggests not replacing conventional PMPR with the use of alternative/adjunctive methods, like Er:YAG laser treatment, sub-antimicrobial dose doxycycline, and photodynamic therapy.
Rapid review (solo researcher)	Routine professional mechanical plaque removal (PMPR) to remove calculus using instrumentation [9]. Can utilize universal curettes (4L-4R) to remove supragingival calculus [10]. Deliver oral hygiene instruction [9]. Risk factor assessment and management [9]. Er:YAG lasers may be used as adjuncts to reduce overall plaque levels [11,15].
ChatGPT4 (OpenAI)	Scaling and Polishing Procedure: Use of dental instruments, like scalers and curettes, to manually remove the calculus. Ultrasonic Scaling: Utilizes high-frequency vibrations to break up calculus. Polishing: Follows scaling to smooth the tooth surface. Patient Education Oral Hygiene Instruction: Educate patients about proper brushing and flossing techniques to prevent the recurrence of calculus. Dietary Advice: Discuss the role of diet in dental health, particularly in the formation of calculus. Regular Dental Check-ups Monitoring: Regular dental visits for professional cleaning and monitoring of oral hygiene. Adjunctive Treatments Fluoride Treatments: Can help strengthen enamel and prevent tooth decay. Mouth Rinses: Antimicrobial mouth rinses can help maintain oral hygiene.
ChatGPT3.5 (OpenAI)	Remove supragingival calculus with scaling and root planing (SRP) through hand scalers, curettes, and ultrasonic scalers. The use of prophylaxis is also recommended. Supplement with oral hygiene instructions, diet, and lifestyle assessment, including tobacco use. Regular maintenance is required.
Bard (Google AI)	Based on systematic reviews, supragingival calculus is effectively treated with SRP. This is recommended if supragingival calculus is evident on a photograph of a patient’s teeth. For heavy calculus, hand scalers are effective, and for light calculus and staining with ultrasonic scalers and air-polishing devices are effective. Deliver oral hygiene instructions. Patients should be instructed on good oral hygiene practices to prevent the buildup of plaque and calculus.

**Table A2:** Tabulated outputs for the treatment of subgingival calculus according to five tools, namely, Delphi group, rapid review, ChatGTP4, ChatGTP3.5, and Bard.

Delphi group (CoTreat AI)	Hand- or power-driven subgingival instrumentation (non-surgical). A. EFP recommends subgingival periodontal instrumentation with hand or powered (sonic/ultrasonic) instruments, either alone or in combination, to treat periodontitis in order to reduce probing pocket depths, gingival inflammation, and the number of diseased sites. The procedure can be performed with either traditional quadrant-wise or full-mouth delivery within 24 h. B. EFP suggests not to use lasers, antimicrobial photodynamic therapy (aPDT), local administration of statin gels (atorvastatin, simvastatin, rosuvastatin), probiotics, systemic sub-antimicrobial dose doxycycline (SDD), locally delivered bisphosphonate (BP) gels, systemic BPs, systemic or local non-steroidal anti-inflammatory drugs (NSAIDs), omega−3 PUFAs, or the local administration of metformin gel as adjuncts to subgingival instrumentation. C. Adjunctive antiseptics may be considered, specifically chlorhexidine mouth rinses for a limited period of time or locally administered sustained-release chlorhexidine, in periodontitis therapy, as adjuncts to mechanical debridement, in specific cases (medical status, adverse effects—staining, economic status). D. Antibiotic use: Specific locally administered sustained-release antibiotics as an adjunct to subgingival instrumentation in patients with periodontitis may be considered. Due to concerns about patient’s health and the impact of systemic antibiotic use on public health, its routine use as an adjunct to subgingival debridement in patients with periodontitis is not recommended. The adjunctive use of specific systemic antibiotics may be considered for specific patient categories (e.g., generalized periodontitis Stage III in young adults).
Rapid review (solo researcher)	Routine professional mechanical plaque removal (PMPR) to remove calculus using instrumentation [9]. Manual subgingival scaling with Gracey curettes is more effective for 4–6 mm pocket depths [12]. Deliver oral hygiene instruction [9]. Risk factor assessment and management [9]. No additional benefit of using antibiotics or photodynamic therapy with mechanical debridement [13].
ChatGPT4 (OpenAI)	Scaling and Root Planing (SRP): The primary treatment for subgingival calculus is scaling and root planing. This deep cleaning procedure involves the removal of plaque and calculus from the tooth surface and root. SRP is performed under local anesthesia and can be conducted using hand instruments (like curettes) and/or ultrasonic scalers. Ultrasonic Scaling: Ultrasonic scalers are often effective at removing subgingival calculus, especially for larger deposits. These devices use high-frequency vibrations to break up calculus and disrupt bacterial biofilms. Adjunctive Therapies: In some cases, adjunctive therapies, such as local delivery antimicrobials or systemic antibiotics, may be considered, especially in the presence of periodontal pockets or active periodontal disease. Patient Education and Oral Hygiene: Educating patients about the importance of oral hygiene in preventing the recurrence of subgingival calculus is crucial. Instruction on proper brushing and interdental cleaning techniques is essential. Regular Maintenance and Follow-Up: Regular periodontal maintenance, including professional cleanings and evaluations, is important to monitor the patient’s periodontal status and prevent the recurrence of calculus. Risk Factor Management: Management of risk factors such as smoking, diabetes, and other systemic conditions that can impact periodontal health is an important part of treatment.
ChatGPT3.5 (OpenAI)	Complete a periodontal assessment and confirm subgingival calculus presence and extent on a radiograph. The gold standard treatment for subgingival calculus is SRP. Hand instruments, with scalers and curettes, or ultrasonic instruments may be used under local anesthetic. If there is active infection or severe periodontal disease, consider adjunctive use of systemic or locally administered antibiotics. Regular periodontal assessment and oral hygiene instructions are required.
Bard (Google AI)	Calculus appears as a radiopaque (white) irregularity at the cementoenamel junction or extending beyond or superimposed over the root surface contour. According to a 2020 systematic review, SRP reduces periodontal pocket depths and improves clinical attachment levels in periodontitis patients. Evidence also suggests that SRP is more effective than oral hygiene instruction alone or the use of adjunctive antimicrobial therapy. According to The European Federation of Periodontology (EFP), SRP is recommended for subgingival calculus treatment in periodontitis patients in all teeth with loss of periodontal support and/or periodontal pockets.
