# Supplementary material for: Accuracy of Treatment Recommendations by Pragmatic Evidence Search and Artificial Intelligence: An Exploratory Study
Source: Diagnostics (Basel). 2024 Mar 1;14(5):527. doi: 10.3390/diagnostics14050527 (PMC10930878; doi:10.3390/diagnostics14050527)
Supplement: Supplementary file 1 [file diagnostics-14-00527-s001.zip › diagnostics-2830825-supplementary.pdf]

SUPPLEMENTARY MATERIAL

Accuracy of treatment recommendations by pragmatic systematic search and artificial intelligence: an exploratory study

Zunaira Baig, Daniel Lawrence, Mahen Ganhewa, Nicola Cirillo

Supplementary Table S1. Search Strategy from Ovid Medline……………….page 2

Supplementary Table S2. Supragingival Calculus…………………………..…page 3

Supplementary Table S3. Subgingival calculus…………………………………page 4

Supplementary Figure S1……………………………………..……………..page 5

References……………………………………..…………………..……….page 6

Supplementary Table S1. Search Strategy from Ovid Medline.

Dental condition	Search strategies	Definition used
1.1 Supragingival calculus	Diagnosis: (“supragingival calculus” or (“supragingival” and “calculus”)) and (“exam*” or “detect*” or “diagnosis*” or “photo*”)	The presence of supragingival calculus observed through photograph.
1.2 Subgingival calculus	Diagnosis: (“subgingival calculus” or (“subgingival” and “calculus”)) and (“radiograph*” or “bitewing” or “BW” or “periapical” or “PA” or “exam*” or “detect*” or “diagnosis*”)	Radiopaque irregularity at cementoenamel junction or extending beyond or superimposed over the root surface contour on PA or BW radiograph.

Supplementary Table S2. Supragingival Calculus.

	Author	Year	Research design	Clinical Examination required (Yes/No/ Not specified)	Observation Image type	Diagnostic category	Treatment options
1	Sanz M, Herrera D, Kebschull M, Chapple I, Jepsen S, Beglundh T, et al.	2020	Clinical Practice Guideline	Yes	Photograph	Visual detection(1)	Routine professional mechanical plaque removal (PMPR) to remove supragingival calculus using instrumentation.<br>Deliver oral hygiene instructions.<br>Risk factor assessment and management(1).
2	Kuka GI, Kuru B, Gursoy H.	2023	RCT	Not specified	Photograph		Can utilise universal curettes (4L-4R) to remove supragingival calculus. This results in smoother surface morphology(2).
3	Gao YZ, Li Y, Chen SS, Feng B, Wang H, Wang Q.	2021	RCT	Not specified	Photograph		Er:YAG lasers may be used as an adjunct with subgingival curettage. This reduces plaque index levels(3).

Supplementary Table S3. Subgingival calculus.

	Author	Year	Evidence type	Clinical Examination required (Yes/No)	Observation image type	Diagnostic category	Treatment options
4	Buchanan SA, Jenderseck RS, Granet MA, Kircos LT, Chambers DW, Robertson PB.	1987			Periapical radiograph	Radiographic detection. Periapical radiograph may be used (4).	
1	Sanz M, Herrera D, Kebschull M, Chapple I, Jepsen S, Beglundh T, et al.	2020	Clinical Practice Guideline	Yes			Routine professional mechanical plaque removal (PMPR) to remove subgingival calculus using instrumentation.<br>Deliver oral hygiene instruction.<br>Risk factor assessment and management(1).
5	Zhang X, Hu Z, Zhu X, Li W, Chen J.	2020	Systematic review				Manual subgingival scaling with Gracey curettes is more effective for 4-6mm pocket depths(5).
6	Manresa C, Sanz-Miralles EC, Twigg J, Bravo M.	2018	Systematic review				No additional benefit to use antibiotics or photodynamic therapy with mechanical debridement (6).

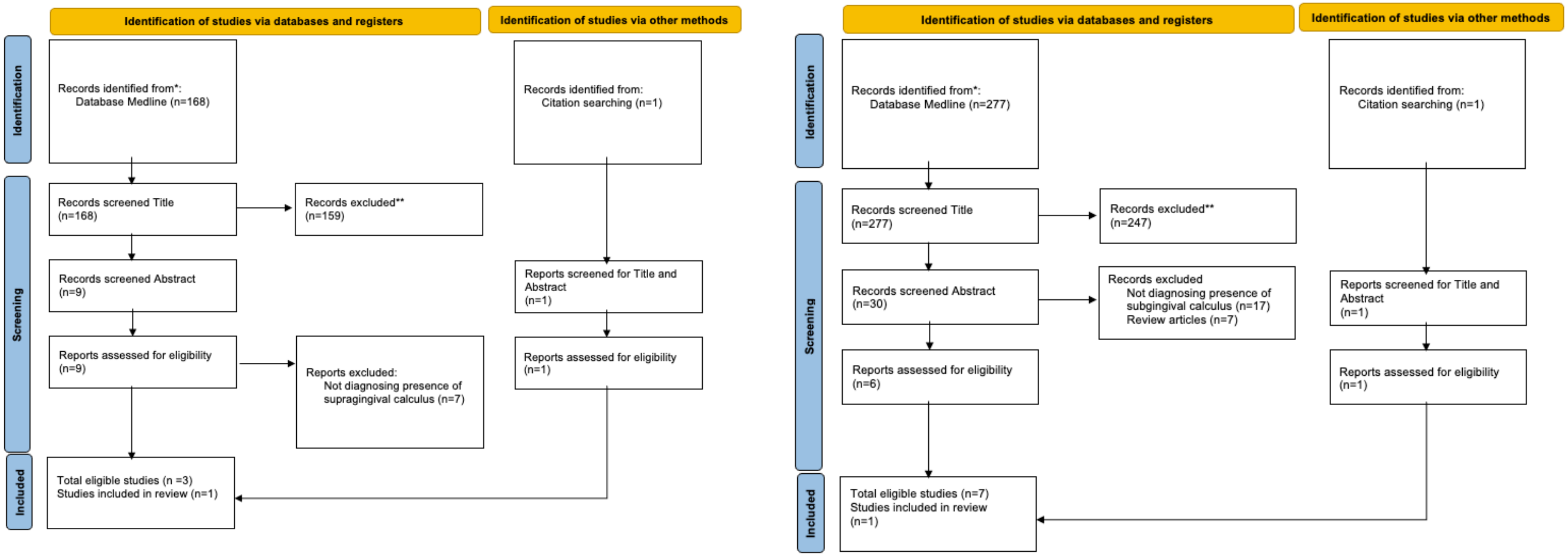

Supplementary Figure S1. PRISMA 2020 for systematic reviews, which included searches of databases, registers and other sources for the diagnosis of supragingival calculus and subgingival calculus.

References

1. Sanz, M.; Herrera, D.; Kebschull, M.; Chapple, I.; Jepsen, S.; Beglundh, T., et al. Treatment of stage I-III periodontitis-The EFP S3 level clinical practice guideline. J *Clin Periodontol*. **2020**, *47* (Suppl 22), 4-60.
2. Kuka, G.I.; Kuru, B.; Gursoy, H. In Vitro Evaluation of the Different Supragingival Prophylaxis Tips on Enamel Surfaces. *Photobiomodul Photomed Laser Surg.* **2023**, *41*, 212-217.
3. Gao, Y.Z.; Li, Y.; Chen, S.S.; Feng, B.; Wang, H.; Wang, Q. Treatment effects and periodontal status of chronic periodontitis after routine Er:YAG laser-assisted therapy. *World J Clin Cases*. **2021**, *9*, 9762-9769.
4. Buchanan, S.A.; Jenderseck, R.S.; Granet, M.A.; Kircos, L.T.; Chambers, D.W.; Robertson, P.B. Radiographic detection of dental calculus. *J Periodontol.* **1987**, *58*, 747-751.
5. Zhang, X.; Hu, Z.; Zhu, X.; Li, W.; Chen, J. Treating periodontitis-a systematic review and meta-analysis comparing ultrasonic and manual subgingival scaling at different probing pocket depths. *BMC Oral Health*. 2020, 20, 176.
6. Manresa, C.; Sanz-Miralles, E.C.; Twigg, J.; Bravo, M. Supportive periodontal therapy (SPT) for maintaining the dentition in adults treated for periodontitis. *Cochrane Database Syst Rev.* **2018**, *1*, CD009376.
7. ChatGPT [Computer Software] [Internet]. 2023. Available online: http://chat.openai.com (6 October 2023).
8. Bard [Computer Software] [Internet]. 2023. Available online: http://bard.google.com (6 October 2023)..
